# A Medical Journalist in Trouble

**Published:** 1898-02

**Authors:** 


**A Medical Journalist in Trouble.**—We see by the *Washington Post* that Dr. W. C. Boteler, editor and publisher of the *North American Medical Review*, has been arrested in Washington City on a charge of criminal libel. It seems the doctor is practicing medicine in Washington, but publishes his journal in Kansas City. The charge is brought by a representative of the H. K. Mulford Co., who alleges that Dr. B., in the pages of his journal, disparaged their *serum*, because, as it is alleged, they wouldn't advertise in his journal, and that the doctor had threatened to make trouble. It seems that he carried out his threat, and fell heir to the "trouble" himself.

